# Reactive Sulfur Species Omics Analysis in the Brain Tissue of the 5xFAD Mouse Model of Alzheimer’s Disease

**DOI:** 10.3390/antiox12051105

**Published:** 2023-05-16

**Authors:** Ayaka Kinno, Shingo Kasamatsu, Takaaki Akaike, Hideshi Ihara

**Affiliations:** 1 Department of Biological Chemistry, Graduate School of Science, Osaka Metropolitan University, Osaka 599-8531, Japan; su23029l@st.omu.ac.jp (A.K.); kasamatsu@omu.ac.jp (S.K.); 2 Department of Environmental Medicine and Molecular Toxicology, Graduate School of Medicine, Tohoku University, Sendai 980-8575, Japan; takaike@med.tohoku.ac.jp

**Keywords:** reactive sulfur species, Alzheimer’s disease, oxidative stress, protein polysulfide, omics

## Abstract

Alzheimer’s disease (AD) is a progressive neurodegenerative disorder whereby oxidative stress augmentation results in mitochondrial dysfunction and cell death by apoptosis. Emerging evidence indicates that reactive sulfur species (RSS), such as glutathione hydropersulfide (GSSH), is endogenously produced, functions as potent antioxidants, and regulate redox signaling through the formation of protein polysulfides. However, the relationship between RSS and AD pathogenesis is not fully understood. In this study, we analyzed endogenous RSS production in the brain tissue of a familial AD model (5xFAD) mouse using multiple RSS-omics approaches. Memory impairment, increased amyloid plaques, and neuroinflammation have been confirmed in 5xFAD mice. Quantitative RSS omics analysis revealed that the total polysulfide content was significantly decreased in the brains of 5xFAD mice, whereas there was no significant difference in the levels of glutathione, GSSH, or hydrogen sulfide between wild-type and 5xFAD mice. In contrast, a significant decline in the protein polysulfide status was observed in the brains of 5xFAD mice, suggesting that RSS production and subsequent redox signaling might be altered during the onset and progression of AD. Our findings have important implications for understanding the significance of RSS in the development of preventive and therapeutic strategies for AD.

## 1. Introduction

Alzheimer’s disease (AD) is one of the neurodegenerative diseases of the brain that is most closely related to aging. AD is characterized by progressive memory impairment and cognitive dysfunction and represents the most frequent form of dementia worldwide; the number of patients with AD is continuously increasing, especially in today’s aging society [1]. Although effective preventive and/or therapeutic strategies are urgently needed, the detailed pathological mechanisms underlying AD remain unclear. One of the most popular hypotheses regarding the underlying mechanism of AD onset is the formation of amyloid beta (Aβ) plaque [2,3]. It has been demonstrated that abnormally elevated levels of oxidative stress markers are associated with AD, suggesting that oxidative stress plays a crucial role in the pathogenesis of this neurodegenerative disorder. It is well established that the extracellular accumulation of Aβ causes the formation of reactive oxygen species (ROS) via mitochondrial dysfunction [4,5]. In addition, nitric oxide (NO) and reactive nitrogen species, including peroxynitrite, are important factors in oxidative stress, leading to detrimental effects on AD onset [6,7,8]. However, it is still a matter of debate and speculation whether impairment of NO/ROS signaling to cause oxidative stress precedes the pathological accumulation of Aβ or vice versa.

Hydrogen sulfide (H_2_S) is known for its broad spectrum of bioactivities as a gaseous signaling molecule, such as the regulation of neurotransmission and the elimination of ROS [9]. Previous studies have reported a severe decline in H_2_S levels in the brain tissue of patients with AD. Additionally, studies have shown that the administration of H_2_S donors effectively prevents AD-induced cognitive dysfunction [10,11]. These observations suggest that H_2_S or related sulfur-containing substances play a crucial role in the pathological progression of AD. Treatment with high concentrations of H_2_S induces mitochondrial dysfunction, causing oxidative stress [12]. This indicates that H_2_S has a dual nature, and consequently, the details of its physiological and pathological activity are unclear. Concerning the biological functions of H_2_S, the endogenous production of reactive sulfur species (RSS), such as cysteine hydropersulfide (CysSSH) and glutathione hydropersulfide (GSSH), has been identified [13]. Of note, H_2_S can be generated via hydrolysis of RSS under physiological conditions, which is enhanced in the presence of electrophilic substances [14,15,16,17]. To accurately detect the polysulfide content in biological samples, our research group developed various quantitative detection methods using liquid chromatography-electrospray ionization-tandem mass spectrometry (LC-ESI–MS/MS) and a stable isotope-labeled standard dilution method [13,16,18,19]. A previous study using our novel alkylating agent, *N*-iodoacetyl l-tyrosine methyl ester (TME-IAM), which can alkylate RSS without artificial decomposition, revealed that the endogenous H_2_S level detected using TME-IAM was significantly lower than that detected using the prototype alkylating agent, β-(4-hydroxyphenyl)ethyl iodoacetamide, in the mouse brain tissue [16]. Thus, controversies have arisen regarding previously reported H_2_S biology [20,21]. 

Our recent study has revealed that cysteinyl-tRNA synthetase (CARS) can produce CysSSH using cysteine as an enzymatic substrate, and other types of RSS, including GSSH, can be generated through a sulfur transfer reaction from the formed CysSSH [18]. Thus, cysteine is an important molecule for endogenous RSS production. In addition, the CysSSH that is formed by CARS and related RSS plays crucial roles in mitochondrial biogenesis and energy metabolism [18,22]. Moreover, it is well established that the RSS is a strong antioxidant and regulates redox signaling through its unique chemical properties as both a nucleophile and an electrophile [13,23]. RSS have been shown to exert protective effects against oxidative stress-related pathological conditions such as methylmercury-induced cytotoxicity, ischemic neurodegeneration, ferroptosis, and lipopolysaccharide-induced inflammatory responses [22,24,25,26]. Considering the previously mentioned pathological impacts of oxidative stress on the onset of AD [4,5,6,8], it is plausible to suggest that an alteration in RSS-dependent redox homeostasis may be involved in the pathological progression of AD. However, the relationship between RSS and AD progression has not yet been established. In this study, we aimed to analyze the endogenous production of RSS in the brain tissue of a well-established mouse model of AD (B6SJL-Tg6799 strain, 5xFAD [27]) using multiple RSS-omics approaches.

## 2. Materials and Methods

### 2.1. Materials

Dithiothreitol (DTT), *N*-ethylmaleimide (NEM), and Blocking One were purchased from Nacalai Tesque (Kyoto, Japan). Biotin-maleimide (BM) was obtained from Sigma-Aldrich (St. Louis, MO, USA). An authentic stable isotope-labeled standard of the bis-sulfur-NEM-adduct (NEM-^34^S-NEM-d_10_) was prepared using stable isotope-labeled NEM (NEM-d_5_) and sodium sulfide (Na_2_^34^S), as previously reported [28]. TME-IAM and authentic stable isotope-labeled GSH/GSSH and H_2_S-adducts of TME-IAM (d_2_) were prepared as previously reported [16]. All other chemicals and reagents were procured from common suppliers and were of the highest commercially available grade.

### 2.2. Animals

5xFAD mice (B6SJL-Tg6799 strain originating from [27]) were obtained from The Jackson Laboratory, and 6 to 8-month-old male mice were used in this study. This study was performed in accordance with the Guidelines for Animal Experimentation of Osaka Metropolitan University, Osaka, Japan. All animal experiments were approved by the Animal Ethics Committee of Osaka Metropolitan University (protocol code: No. 2022-95, date of approval: 1 April 2022). The animals were reared at 22–24 °C with a 12 h light/12 h dark cycle. The animals were fed commercial pellets (CE-2; CLEA Japan Inc., Tokyo, Japan) and provided with water ad libitum. The genotype of individual animals was determined by polymerase chain reaction, as recommended by the Jackson Laboratory. Additionally, homozygous wild-type (WT) and heterozygous 5xFAD mice were used in this study. The mice were anesthetized by isoflurane inhalation and euthanized by cervical dislocation. The brain tissue was immediately removed, and the cerebral cortex was harvested. After harvesting, the cerebral cortex was frozen in liquid nitrogen and stored at -80 °C until the following analysis.

To obtain rat anti-TME-IAM antiserum, a 10-week-old male Wistar rat (Kiwa Laboratory Animals, Wakayama, Japan) was subcutaneously administered TME-IAM-treated bovine serum albumin (BSA). The TME-IAM-treated BSA was prepared by incubating BSA in the presence of TME-IAM at neutral pH for 1 h plus Freund’s complete adjuvant (Difco Laboratories, Detroit, MI, USA). Booster doses of the same immunogen plus Freund’s incomplete adjuvant (Difco Laboratories) were administered three times every two weeks. Three days after the last booster dose, the immunized rats were anesthetized with isoflurane and sacrificed by collecting the whole blood volume, followed by serum separation. The reactivity of rat serum against the TME-IAM-modified proteins was confirmed by ELISA using TME-IAM-treated chicken ovalbumin.

### 2.3. Evaluation of Cognitive Function by a Behavior Analysis Test (Y-Maze Test)

The cognitive function of mice was evaluated using the Y-maze test, as previously reported, with minor modifications [29,30]. Prior to the test, each mouse was allowed to freely explore the experimenter’s hand for 2 min twice per day for 2 days to minimize the influence of the experimenter on the mice. On the day of the test, each mouse was once again acclimated to the experimenter’s hands and then placed in the center of the Y-maze (SHINFACTORY, Fukuoka, Japan). The sequence of arm entries over 8 min was recorded on a video. An arm entry was completed when the head and hind paws of the mouse had been completely 5 cm into the arm, and entries into three different arms (e.g., A/B/C or C/B/A) were recorded as correct alternations. The following equations were used to calculate the alternating behavior performance:

Correct alternation rate (%) = number of correct alternations/(total alternations − 2)

In contrast, the number of arm entries was considered the spontaneous behavior of the mouse.

### 2.4. Imaging Analysis

After perfusion–fixation of mice under isoflurane inhalation, brains were extracted, and 20 µm thick frozen sections were prepared using a cryostat (Leica Biosystems, Wetzlar, Germany). The sections were permeabilized by washing three times for 5 min in phosphate-buffered saline (PBS) containing 0.3% TritonX-100 (PBST) and then blocked for 2 h at room temperature in PBST containing 1% BSA. The brain sections were then incubated overnight at 4 °C with rabbit anti-ionized calcium-binding adapter molecule-1 (Iba1) (Fujifilm Wako, Osaka, Japan) and mouse anti-glial fibrillary acidic protein (GFAP)-Cy3 (Sigma-Aldrich), diluted 1000- and 400-fold, respectively, in PBST containing 1% BSA. After washing with PBST three times, the sections were incubated in Can Get Signal Solution II (Toyobo, Osaka, Japan) containing Amylo-Glo (Biosensis, Thebarton, Australia), a fluorescent probe for Aβ plaque, and 500-fold-diluted goat-anti-rabbit-Cy5 (AnaSpec, Fremont, CA, USA) at room temperature for 2 h. Thereafter, the brain sections were washed three times for 5 min with PBST and sealed in VECTASHIELD Mounting Medium (Vector Laboratories, Newark, CA, USA). An all-in-one fluorescence microscope (BZ-X800; Keyence, Osaka, Japan) with BZ-X800 filter DAPI for Amylo-Glo, BZ-X800 filter green fluorescent protein for GFAP, and BZ-X800 filter Cy5 for Iba1 was used to detect signals.

### 2.5. Quantification of the Total Polysulfide Content Using Alkaline/Reductive Sulfur Elimination Protocol

The total polysulfide content (TPsC) was quantified using a recently developed method [19]. Each cerebral cortex of a mouse (22–47 mg) was homogenized in a 20-fold volume of 100 mM *N*-cyclohexyl-3-aminopropanesulfonic acid-NaOH buffer (pH 11.0) containing 10 mM DTT and 25% (*v*/*v*) ethanol. The resulting solution was incubated at 37 °C for 1 h to decompose RSS into sulfide anions. The reaction solution was then diluted 10-fold in 200 mM Tris (hydroxymethyl)aminomethane (Tris)-HCl buffer (pH 7.4) containing 20 mM NEM and 80% (*v*/*v*) methanol and incubated at 37 °C for 1 h to convert sulfide anions to NEM-S-NEM. After centrifugation, the supernatant was diluted 10-fold with 0.1% (*v*/*v*) formic acid (FA) solution containing 0.1 μM isotope-labeled standard NEM-^34^S-NEM-d_10_, and LC-ESI-MS/MS analysis was performed as described in Section 2.10.

### 2.6. Quantification of the Total Sulfur Content by Acid-Circulating Decomposition and Inductively Coupled Plasma Optical Emission Spectroscopy

The total sulfur content (TSC) in the mouse cerebral cortex was quantified using a recently developed method [19]. Briefly, the cerebral cortex (10–60 mg) was subjected to an acid-circulating decomposition system (ECOPRE system; Actac, Tokyo, Japan) with multiple heating/cooling cycles on a graphite hot plate (Asone, Osaka, Japan). The resulting brain homogenate was filled up to 30 mL with 1 M HNO_3_, and the concentration of sulfate ions in the sample was measured using inductively coupled plasma optical emission spectroscopy (ICP-OES) (ICPE-9000; Shimadzu, Kyoto, Japan) with a standard curve of Sulfur Standard Solution (Kanto Chemical, Tokyo, Japan). The ICP-OES measurement parameters were set as follows: argon gas pressure 450 ± 10 kPa, RF power 1.2 kW, plasma gas flow rate 14 L/min, carrier gas flow rate 0.7 L/min, exposure time 30 s, and observation wavelengths 180.731, 182.037, and 182.625 nm.

### 2.7. Evaluation of RSS-Producing Enzyme Expression Levels by Western Blotting

The mouse cerebral cortex was lysed in a lysis buffer containing 50 mM 4-(2-hydroxyethyl)-1-piperazineethanesulfonic acid (HEPES)-NaOH (pH 8.0), 150 mM NaCl, 10 mM sodium pyrophosphate, 10 mM sodium fluoride, 2% (*v*/*v*) 3-[(3-Cholamidopropyl)dimethylammonio]propanesulfonate (CHAPS), 2.5% lithium dodecyl sulfate, 10% (*v*/*v*) glycerol, 2 mM ethylenediaminetetraacetic acid (EDTA), 2 mM sodium vanadate, protease inhibitor cocktail (Nacalai Tesque), and 1 mM DTT. Samples were separated by SDS-PAGE and transferred to nitrocellulose membranes. The membranes were blocked using Blocking One and then incubated overnight at 4 °C with primary antibodies, including anti-CARS1 antibody (1:1000 dilution; Merck Millipore, Burlington, MA, USA, rabbit), anti-CARS2 (1:1000 dilution; [18], rat), anti-cystathionine β-synthase (CBS) (1:1000 dilution; Abnova, Taipei, Taiwan, mouse), and anti-cystathionine γ-lyase (CSE) (1:1000 dilution; [31], rabbit) in Can Get Signal Solution I (Toyobo). After washing with 0.05% Tween 20-containing Tris-buffered saline (TBST) containing 50 mM Tris-HCl (pH 7.5), 137 mM NaCl, and 2.68 mM KCl, the membranes were incubated with anti-rabbit peroxidase (POD) (Santa Cruz Biotechnology, Dallas, TX, USA), anti-mouse POD (Cytiva, Tokyo, Japan), and anti-rat POD (Cell Signaling Technology, Danvers, MA, USA) in TBST for 1 h at room temperature. After washing with TBST, the samples were treated with Immobilon Forte Western HRP Substrate (Merck Millipore), and signals were detected using a luminescent image analyzer (LAS-1000 mini; Fujifilm). Signal intensities were analyzed using analysis software (Multigauge, Fujifilm). The uncropped Western blotting images are provided in the original Western blotting Figure S1.

### 2.8. Quantitative Sulfur Metabolomics Using LC-ESI–MS/MS with TME-IAM

Endogenous production of RSS, including GSSH, GSH, H_2_S, and cysteine, was detected using quantitative metabolomics, as described previously [16]. Moreover, oxidized glutathione disulfide (GSSG) was detected as a marker of oxidative stress by LC-ESI–MS/MS. Briefly, cerebral cortex tissues (16–47 mg) were homogenized in a 20-fold volume of 50 mM sodium acetate buffer (pH 6.5) containing 1 mM TME-IAM and 70% methanol and then incubated at 37 °C for 30 min. After centrifugation at 20,380× *g* at 4 °C for 15 min, 100 µL of the supernatant was acidified with 10 µL of 10% FA and diluted 10-fold with a stable isotope-labeled standard. The resulting solution was then subjected to centrifugal filtration at 1000× *g* at 4 °C for 1 min, and the elution was applied to LC-ESI–MS/MS analysis. MRM parameters are provided in Supplementary Table S1.

### 2.9. TME-IAM Switching Assay

Mouse cortical tissue was homogenized in a 20-fold volume of RIPA buffer containing 10 mM Tris-HCl (pH 7.4), 150 mM NaCl, 0.1% sodium deoxycholate, 0.1% SDS, 1% NP-40, 10 mM trans-Epoxysuccinyl-l-leucylamido(4-guanidino)butane (E-64) (Sigma), 10 mM Bestatin hydrochloride (Sigma), 10 mM Pepstatin A (Peptide Institute, Osaka, Japan), 10 mM Leupeptin (Peptide Institute), and either 1 mM TME-IAM or 3 mM BM. The homogenate was then centrifuged at 20,380× *g* at 4 °C for 15 min and incubated at 37 °C for 1 h while rotating. A part of the TME-IAM-treated samples was then diluted with RIPA buffer containing BM (final concentration 3 mM), and further incubated at 37 ℃ for 1 h while rotating. After boiling in SDS sample buffer in the absence of reductants, proteins were subjected to SDS-PAGE, followed by Coomassie Brilliant Blue (CBB) stain or Western blotting using either Poly-HRP Streptavidin (200,000 dilutions; Thermo Fisher Scientific, Waltham, MA, USA) or rat anti-TME-IAM antiserum (1:3000 dilution). The procedures for signal detection and band intensity quantification were identical to those described in Section 2.7. Protein persulfides were estimated by semi-quantitative analysis of the band intensities of samples treated with both TME-IAM and BM or only BM. The uncropped Western blotting images are shown in the original Western blotting Figure S2.

### 2.10. LC-ESI–MS/MS

LC-ESI–MS/MS analyses were performed using a triple quadrupole mass spectrometer (Xevo TQD; Waters, Milford, MA, USA) and an Alliance e2695 system (Waters). The separation of each adduct was achieved using the Alliance e2695 system equipped with a C18 reverse-phase column (Mightysil RP-18 GP 2.0 × 50 mm, Kanto Chemical) with a linear gradient of solvent A (water containing 0.1% FA) and solvent B (100% methanol) at a flow rate of 0.6 mL/min: detection gradient for NEM adducts: 1% B at 1 min, 99% B at 4 min; detection gradient for TME-IAM adducts: 1% B at 1 min, 99% B at 7 min. The mass spectrometer was operated in positive mode with a capillary voltage of 1000 V, a desolvation gas (N_2_) flow rate of 1000 L/h, and a temperature of 500 °C. Endogenous adducts and spiked stable isotope-labeled standard adducts were simultaneously identified by multiple reaction monitoring (MRM); MRM parameters are shown in Supplementary Table S1.

### 2.11. Statistical Analysis

All experiments were performed at least three times, and values for individual experiments are presented as mean ± standard error (SE). Statistical significance was determined by Student’s unpaired *t*-test using GraphPad Prism 8.1.2 software (GraphPad, Inc., La Jolla, CA, USA), and *p* < 0.05 was considered significant.

## 3. Results

### 3.1. Confirmation of AD Onset in the 5xFAD Mice

**Figure 1:**
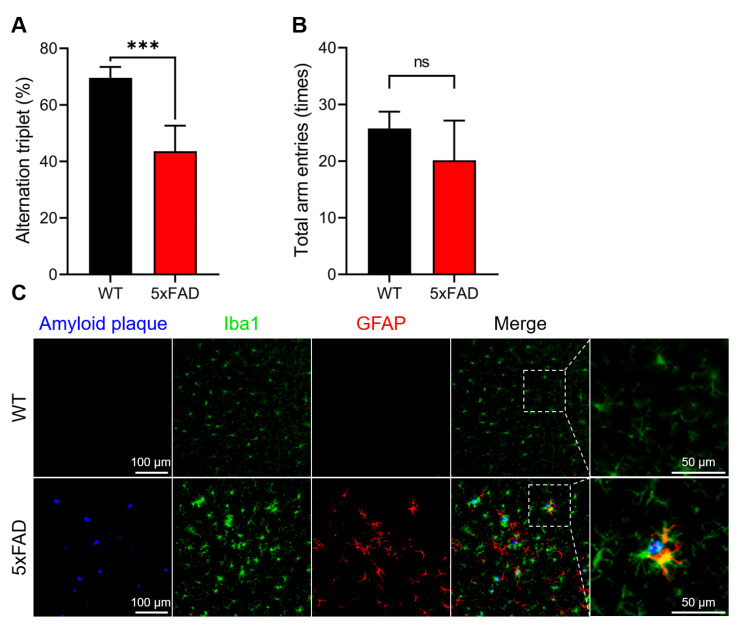
Confirmation of AD onset by the Y-maze test and histochemical analysis. The pathological condition of the model mice of familial Alzheimer’s disease (5xFAD) was evaluated by the Y-maze test (**A**,**B**) and histochemical analysis (**C**). Triple-immunofluorescent demonstration of plaque-associated mixed glial inflammation: activated microglia (green, Iba1) and reactive astroglia (magenta, GFAP) colonizing the periphery of amyloid cores (blue, amyloid plaque) in the cerebral cortex of the 5xFAD mouse brain. Amyloid plaques were detected using a fluorescent probe, Amylo-Glo. Ionized calcium-binding adapter molecule-1 (Iba-1) and glial fibrillary acidic protein (GFAP) were immunochemically detected using specific antibodies. Data are presented as mean ± standard error (SE) (*n* = 4–7). *** *p* < 0.001 versus the wild type (WT) group, compared by Student’s unpaired *t*-test. ns, not significant.

The 5xFAD mouse strain is a well-established model of AD that mimics the major pathological and behavioral characteristics of AD [27]. The pathological conditions of the 5xFAD mice used in this study were evaluated using behavioral (Y-maze test) and histochemical analyses (Figure 1).

As shown in Figure 1A, 5xFAD mice showed significantly impaired spatial working memory in the Y-maze test. However, there was no significant difference in the total arm entries (Figure 1B), indicating that the locomotor functions of these two animal groups were identical. Histochemical analysis revealed a remarkable increase in amyloid plaque formation, Iba-1, and GFAP, which are considered hallmark pathological features of AD and neuroinflammatory markers, respectively, in 5xFAD mice (Figure 1C). Based on these results, we concluded that the 5xFAD mice used in this study had developed AD.

### 3.2. Quantification of the TPsC Using Alkaline/Reductive Sulfur Elimination Protocol and the TSC by Acid-Circulating Decomposition System

**Figure 2:**
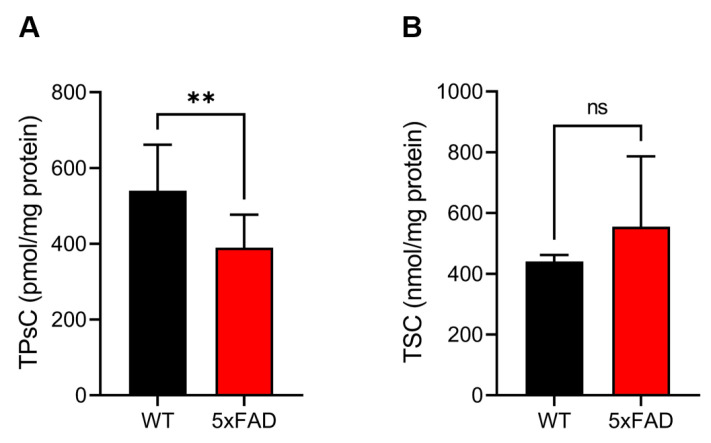
Quantification of the TPsC and TSC. (**A**) The total polysulfide content (TPsC) and (**B**) the total sulfur content (TSC) were determined by the alkaline/reductive sulfur elimination protocol followed by liquid chromatography-electrospray ionization-tandem mass spectrometry (LC-ESI–MS/MS) and acid-circulating decomposition and inductively coupled plasma optical emission spectroscopy, respectively. Data are presented as means ± SE (*n* = 4–13). ** *p* < 0.01 versus the WT group, compared by Student’s unpaired *t*-test. ns, not significant.

In a recent study, we succeeded in developing novel methods to quantitatively determine the TPsC and TSC in biological samples [19]. Thus, we attempted to quantify the TPsC and TSC in the cerebral cortex of 5xFAD mice (Figure 2). As shown in Figure 2A, a significant decrease in the TPsC was observed in 5xFAD mice. In contrast, there was no significant difference in the TSC between the WT and 5xFAD mice (Figure 2B). These results indicate that the amount of polysulfides, but not the amount of sulfur compounds, was significantly reduced in 5xFAD mice.

### 3.3. Evaluation of RSS-Producing Enzyme Expression Levels

**Figure 3:**
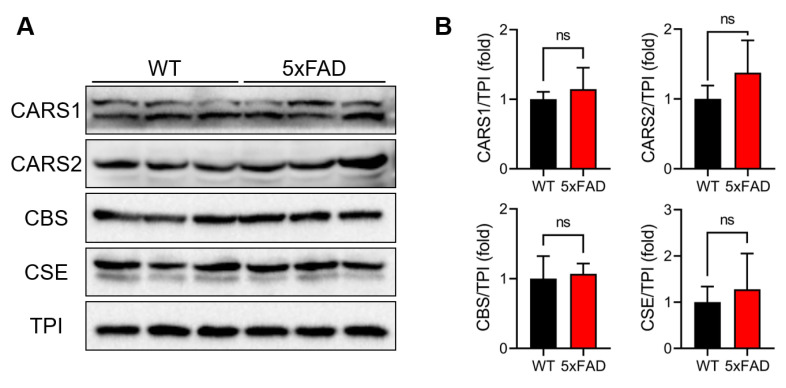
Evaluation of the expression profile of RSS-producing enzymes. (**A**) Representative images of Western blotting with specific antibodies for cytosolic cysteinyl-tRNA synthetase (CARS1), mitochondrial CARS (CARS2), cystathionine β-synthase (CBS), cystathionine γ-lyase (CSE), and triosephosphate isomerase (TPI). (**B**) Semiquantitative presentation of band intensities shown in (**A**). The band intensities were normalized by the band intensity of TPI. Data are presented as mean ± SE (*n* = 5). Statistical analysis was performed with Student’s unpaired *t*-test. ns, not significant.

We previously found that CARS is responsible for the endogenous production of RSS using cysteine as an enzymatic substrate. We identified two types of CARS proteins in mammals, including humans and mice: the cytosolic form (CARS1) and the mitochondrial form (CARS2) [18]. In addition, CBS and CSE are known for their enzymatic activities in producing RSS using cystine, an oxidized form of cysteine, as a substrate [13]. Thus, Western blotting with specific antibodies against these four enzymes was carried out to evaluate their expression profiles in the brain tissue of 5xFAD mice (Figure 3). There were no significant differences in any of the four RSS-producing enzymes between the WT and 5xFAD mice (Figure 3).

### 3.4. Quantitative Sulfur Metabolomics Using LC-ESI–MS/MS with TME-IAM

**Figure 4:**
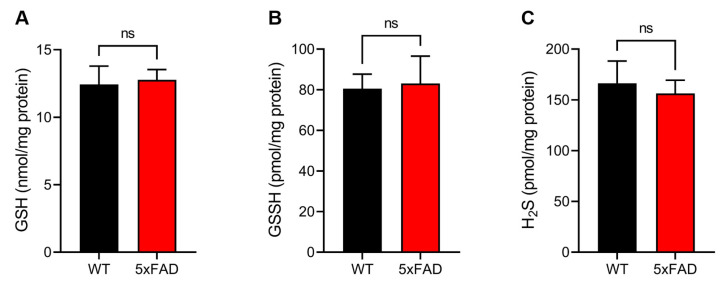
Quantification of RSS metabolites. (**A**) Glutathione (GSH), (**B**) glutathione hydropersulfide (GSSH), and (**C**) hydrogen sulfide (H_2_S) were quantified by LC-ESI-MS/MS with *N*-iodoacetyl l-tyrosine methyl ester (TME-IAM). Data are presented as mean ± SE (*n* = 3). Statistical analysis was performed with the Student’s unpaired *t*-test. ns, not significant.

Previous studies have demonstrated that GSSH is one of the most abundant RSS in vivo [13,16,18,23,32]. Under oxidative stress conditions, GSH is oxidized to produce GSSG, and the GSSG:GSH ratio is recognized as an oxidative stress marker. We performed quantitative metabolomics analysis using LC-ESI–MS/MS with TME-IAM to determine the levels of GSSH and GSH, the precursor molecule of GSSH, as well as H_2_S, cysteine, and GSSG (Figure 4 and Figure S3). As shown in Figure 4, there were no significant differences in the levels of GSH, GSSH, or H_2_S between WT and 5xFAD mice. Similarly, a significant difference in the content of cysteine, an essential substrate for RSS production regulated by CARS, between the WT and 5xFAD mice was not observed (Supplementary Figure S1A). In contrast, the GSSG:GSH ratio in the 5xFAD mice was significantly greater than that in the WT mice (Supplementary Figure S1B). This indicates that oxidative stress increased in the cerebral cortex in the 5xFAD mice. These findings suggest that the significant decrease in the TPsC in the 5xFAD mouse brain might be due to other forms of RSS rather than RSS metabolites. 

### 3.5. Evaluation of Protein Polysulfides by TME-IAM Switching Assay

**Figure 5:**
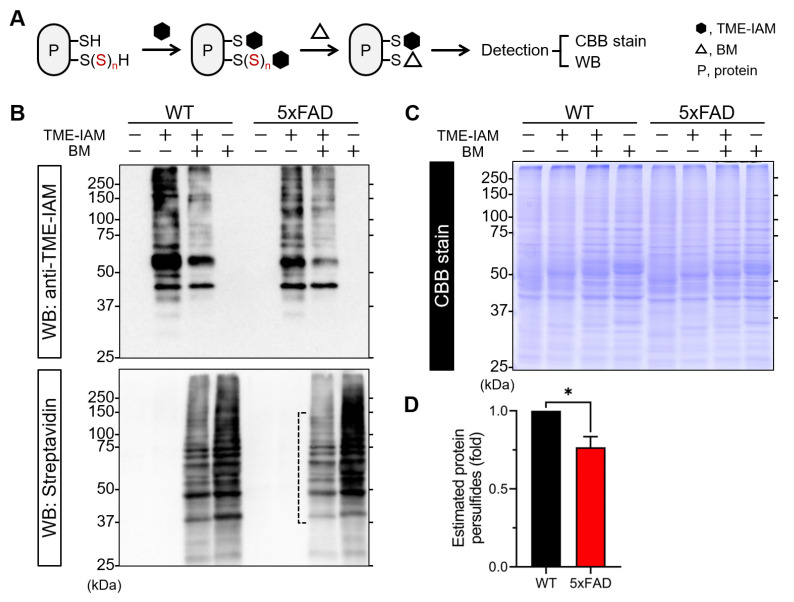
Evaluation of protein polysulfides by the TME-IAM switching assay. (**A**) Overview of the TME-IAM switching assay. Briefly, both normal cysteine thiols (-SH) and polysulfidated cysteine residues [-S(S)_n_H, *n* > 1] on a protein were alkylated with TME-IAM, and a subsequent reaction with biotin-maleimide (BM) resulted in the biotinylation of the polysulfidated cysteine residues. After denaturing in the absence of reductants, proteins were separated by SDS-PAGE. This was followed by Western blotting with rat antiserum specific for TME-IAM ((**B**), **lower**) and Streptavidin ((**B**), **upper**) to detect TME-IAM-labeled and biotinylated proteins, respectively, or Coomassie Brilliant Blue (CBB) stain to visualize protein profiles. (**B**,**C**) Representative images of the respective Western blotting and CBB stain are shown. (**D**) The result of semiquantitative analysis of the band intensities detected by Western blotting with streptavidin. The band intensities of samples treated with both TME-IAM and BM (lanes 3 and 7) were normalized by those of samples treated with only BM (lanes 4 and 8), respectively. The analysis of band intensities detected with streptavidin was performed on the dashed line in Figure ((**B**), **lower**). Data are represented as mean ± SE (*n* = 3). * *p* < 0.05 versus the WT group, compared by Student’s unpaired *t*-test.

Our previous study demonstrated that CARS catalyzes the aminoacylation of tRNA with CysSSH to form CysSSH-conjugated tRNA, which can then be incorporated into a nascent peptide chain. This means that protein polysulfidation can occur during protein translation [18]. Moreover, protein polysulfides can be generated via sulfur transfer reactions with RSS metabolites such as GSSH and a cysteine residue of proteins [33]. Thus, we attempted to detect protein polysulfides in 5xFAD mice using the TME-IAM switching assay (Figure 5). Our previous study revealed that treatment with weak electrophiles, e.g., 8-nitroguanosine 3′,5′-cyclic monophosphate, 2-aminosulfonyl benzothiazole, and β-(4-hydroxyphenyl)ethyl iodoacetamide, can leave protein polysulfides intact as in the original [18,34]. TME-IAM is a superior electrophile to alkylate polysulfidated residues without artificial decomposition of polysulfide structures [16], resulting in the maximization of reaction efficiency between BM and the remaining polysulfide structure to generate biotinylated proteins. As illustrated in Figure 5A, TME-IAM alkylates both normal cysteine thiol residues and polysulfidated cysteine residues on a protein. This is followed by a subsequent reaction with BM, which exhibits a more potent electrophilic property than TME-IAM and can react with the remaining sulfane sulfur atom on the polysulfidated residues. This process leads to the elimination of TME-IAM labeling on polysulfidated residues and the biotinylation of the site. After SDS-PAGE, Western blotting with rat antiserum specific for TME-IAM and streptavidin and CBB stain were carried out to detect TME-IAM labeling and biotinylation of proteins and to visualize protein profiles, respectively. Western blotting with TME-IAM-specific antiserum revealed that TME-IAM labeling was partially eliminated by a subsequent reaction with BM (Figure 5, upper panel). Accordingly, the band intensities of samples treated with both TME-IAM and BM (lanes 3 and 7) were weaker than those of BM-treated samples (lanes 4 and 8) (Figure 5, lower). This suggests that the replacement of TME-IAM labeling with biotinylation occurred at polysulfidated cysteine residues on the respective proteins. Notably, the disappearance of some bands was observed only in the 5xFAD mice. We can evaluate the overall alkylating status of proteins from the band mobility on SDS-PAGE because the alkylation by BM causes an addition of 0.5 kDa to the protein body, while a modification with TME-IAM causes only an addition of 0.2 kDa to the protein body. Indeed, as shown in Figure 5C, the results obtained from the CBB stain showed that the band mobility greatly differed according to the alkylation conditions, particularly in the brain tissue of WT mice. This indicates that the polysulfidation status of cerebral cortex proteins in WT mice may be higher than that in 5xFAD mice. This observation was supported by the results of a semi-quantitative analysis of Western blotting with streptavidin (Figure 5D). Taken together, these results suggest that the levels of polysulfidated proteins in the brain tissue of 5xFAD mice may be lower than those in WT mice. In addition, some of the protein polysulfide bands were found to have partially altered intensities in 5xFAD mice, suggesting that the decrease in these protein polysulfides may play a role in the onset of AD.

## 4. Discussion

In this study, we performed quantitative RSS omics analysis using LC-ESI-MS/MS to investigate RSS production in the cerebral cortex of 5xFAD mice with AD. This analysis revealed a reduction in TPsC and protein polysulfides. To our knowledge, this is the first study to examine the association between RSS and AD in the mouse cerebral cortex.

Over the past two decades, growing evidence has demonstrated that H_2_S is endogenously generated by CBS, CSE, and via 3-mercaptopyruvate sulfurtransferase-mediated cysteine metabolism in mammalian cells and tissues [9,35,36]. H_2_S may function as an important neuromodulator and neuroprotective substance in terms of physiological activities. These include its impact on antioxidant enzymes such as SOD, GPx, and CAT; its antioxidant effects achieved through *S*-sulfhydration of Keap-1 [37,38], its anti-inflammatory effects on LPS and Aβ-induced inflammatory reactions [39], and its anti-apoptotic effects via the inhibition of NF-κB and caspase-3 activation [40]. Therefore, this gaseous signaling molecule has been speculated to be involved in AD for a long time. Indeed, various studies on patients with AD and animal models of AD have reported a severe impairment in the CBS-mediated transsulfuration pathway. These studies have revealed the downregulation of CSE expression and downstream signaling pathways in the plasma and brain tissue, resulting in an abnormal accumulation of homocysteine and a severe decline in H_2_S levels [10,41,42,43]. Furthermore, through in vitro and in vivo experiments, the protective and preventive effects of treatment with exogenous sulfur-containing compounds have been demonstrated. These compounds include diallyl trisulfide, which is a predominant component in garlic essential oils, as well as H_2_S donors, including sodium hydrogen sulfide and sodium sulfide, and H_2_S-releasing compounds such as GYY4137. These studies suggest that H_2_S acts as a potential therapeutic compound for AD in several models, possibly by interfering with multiple targets [37,44,45,46]. However, as mentioned earlier, the details of the physiological and pathological activity of hydrogen sulfide are uncertain due to experimental limitations [14,15,16,17,18,19,20,21]. Moreover, the methods used to measure H_2_S have proven controversial, and it is difficult to compare the values obtained from these measurements [21]. Thus, the precise molecular mechanism of action of H_2_S in AD is not fully understood. Recent studies have shown that H_2_S can be produced via the degradation of polysulfides [14,15,16,18]. Therefore, in the field of sulfur biology, it is quite important to choose an appropriate method to precisely detect individual sulfur-containing molecules, including RSS such as GSSH.

In the current study, we measured the TPsC and TSC using our novel methods, namely the alkaline/reductive sulfur elimination protocol followed by quantitative LC-ESI-MS/MS analysis, acid-circulating decomposition, and ICP-OES, both of which have been developed recently [19]. Our previous study demonstrated that the sulfur atom(s) in all analyzed sulfur-containing substances, including volatile garlic polysulfides such as diallyl tri- or tetrasulfide and hydrophilic polysulfides such as oxidized glutathione tri- or tetrasulfide, undergo complete oxidation to sulfate anions through acid-circulating decomposition. This specific oxidation process was successfully detected using ICP-OES [19]. In contrast, the alkaline/reductive sulfur elimination protocol can only detect the sulfane sulfur atom(s) present in the polysulfides. This protocol involves the reduction of the polysulfides using DTT, followed by the alkylation of the released sulfur atom(s) with NEM, resulting in the formation of a NEM-adduct of sulfur. The formation of the NEM-adduct of sulfur does not occur from the sulfur atom(s) in sulfides (dimethyl sulfide and diallyl sulfide), disulfides (dimethyl disulfide, diallyl disulfide, and oxidized glutathione disulfide), or the thiol (glutathione) [19]. Thus, using these two methods, it is possible to analyze the polysulfide profiles of biological samples. The results obtained in this study showed that there was no significant difference in TSC between the cerebral cortex of WT and 5xFAD mice, whereas 5xFAD mice exhibited a significantly lower TPsC value than WT mice. These results suggest that the overall amount of sulfur atoms in brain tissue may not be affected by the onset of AD. However, the qualitative nature of the forms of sulfur-containing molecules, such as polysulfides, can be altered by the onset of AD. Furthermore, the use of TME-IAM in quantitative RSS metabolomic analysis allows for the precise determination of endogenous H_2_S levels as it can derivatize RSS without artificial decomposition. The results obtained using TME-IAM demonstrated that there was no significant difference in the levels of H_2_S and GSSH, the predominant molecule of endogenous RSS, between WT and 5xFAD mice. Together with the decline in the TPsC, these results strongly suggest that other crucial forms of endogenous RSS may be present in vivo rather than H_2_S and RSS metabolites, the levels of which may be altered by the onset of AD. 

RSS, including H_2_S and polysulfides, is known to react with redox-sensitive cysteine residues of proteins, resulting in the formation of persulfide or polysulfidated moieties [-S(S)_n_H, *n* ≧ 2], which has been considered a major pathway of H_2_S and RSS signaling [47,48]. Indeed, a growing number of persulfidated/polysulfidated proteins have been identified using different labeling methods and proteomic approaches [13,18,49,50,51]. Persulfidation and polysulfidation at an important cysteine residue of target proteins may potentially act as regulators of enzymatic functions and signaling processes [13,52,53,54]. In addition, based on their chemical properties, accumulating evidence indicates that these modifications could serve as a means to protect critical cysteine residues against irreversible oxidative inactivation under elevated oxidative stress [33,55,56,57]. Therefore, the role of protein persulfides/polysulfides in oxidative stress-related neurological diseases such as Parkinson’s disease and AD has gained attention as a primary element in the fundamental mechanisms that underlie H_2_S- and RSS-regulated signaling pathways [58,59]. In support of the hypothesis that protein persulfides/polysulfides play a crucial role in the following signaling pathway, the results obtained by the TME-IAM switching assay showed that the levels of protein persulfides/polysulfides were significantly lower in the brain tissue of 5xFAD mice than in WT mice. These results suggest that the protein persulfides/polysulfides-dependent signaling pathway may be impaired by irreversible oxidative modification of critical cysteine residues of target proteins. 

Mitochondria are important organelles for energy production. As mentioned earlier, RSS is generated in mitochondria, and the catabolism of RSS, including CysSSH, plays crucial roles in energy production and metabolism [18,22]. Impairment of NO/ROS signaling, which causes oxidative stress and mitochondrial dysfunction, has been suggested as a potential pathogenetic mechanism of AD [6,7,8]. Mitochondrial abnormalities include altered morphological defects and reduced numbers in the brain tissue of AD and are closely correlated with mitochondrial dysfunctions [60]. Disruption of the balance between mitochondrial fission and fusion can lead to excessive mitochondrial fragmentation. Fragmentation triggered by dysfunction of the fission-inducing protein, dynamin-related protein 1 (Drp1), for example, contributes to synaptic damage and subsequent neuronal loss because of nitrosative/oxidative stress and impaired bioenergetics [61,62,63]. A previous study by Cho et al. reported that NO produced in response to Aβ protein induces *S*-nitrosylation at cysteine^644^ in human Drp1 protein, resulting in pathological mitochondrial fission and neuronal damage [64]. Recent studies have revealed that the enzymatic activity of Drp1 is tightly regulated by protein persulfidation [18,65]. In other words, the persulfidation at cysteine^624^ in the rat Drp1 protein was found to negatively regulate enzymatic activity, resulting in the maintenance of mitochondrial quality and energy metabolism under physiological conditions. Nishiyama et al. also demonstrated that exposure to a low, non-neurotoxic dose of methylmercury causes depersulfidation at the redox-sensitive cysteine residue in neonatal rat cardiomyocytes, accompanied by Drp1 activation and mitochondrial hyperfission [65]. Notably, methylmercury-dependent desulfidation of Drp1 and mitochondrial hyperfission were almost completely abolished by treating cardiomyocytes with NaHS [65]. These results strongly suggest that depersulfidation of redox-sensitive proteins, including Drp1, may be related to the onset and progression of AD. In the current study, Western blotting with streptavidin revealed that the status of protein persulfides in the cerebral cortex of 5xFAD mice differed from that of WT mice. This suggests that the status of endogenous polysulfidated proteins, including mitochondrial proteins that play a crucial role in RSS catabolism and contribute to mitochondrial energy production and metabolism [22,34,66], may be associated with the pathology of AD in the 5xFAD mice. However, whether the alterations in protein polysulfides are preceded by mitochondrial abnormalities or are because of mitochondrial dysfunction remains unclear. Although the novel method is an easy, convenient, and reliable method to detect protein polysulfides, there are limitations to this method. A major limitation of this method is that the comprehensive identification of polysulfidated proteins is difficult. The number of sulfane sulfurs added to the cysteine residues, i.e., the length of the polysulfide structure, and the number of polysulfidated residues in a certain protein cannot be determined by this method. Therefore, further studies incorporating mass spectrometric analyses are required to identify the polysulfidated proteins that are tightly linked to the onset and progression of AD. These studies should also investigate the correlations between the protein polysulfide status and the protein functions, together with analyses of mitochondrial abnormalities, including morphological changes and impaired energy metabolism.

## 5. Conclusions

In conclusion, we found that the total polysulfide content (TPsC) in the cerebral cortex of a mouse model of familial Alzheimer’s disease (5xFAD mice) was significantly lower than that in WT mice, whereas there was no significant difference in the total sulfur content (TSC). Quantitative mass spectroscopy demonstrated that 5xFAD and WT mice had almost the same endogenous levels of hydrogen sulfide (H_2_S), glutathione hydropersulfide (GSSH), which is a predominant reactive sulfur species (RSS), and glutathione (GSH). In contrast, the TME-IAM switching assay showed a decrease in the levels of protein persulfides/polysulfides in 5xFAD mice, accompanied by the observation of a distinct pattern of protein persulfides/polysulfides. Although further studies are required to identify the pathophysiological significance of protein persulfides in the onset and progression of AD, our results indicate that RSS content in the brain is decreased in AD onset, implying that RSS, including protein persulfides/polysulfides, could be a potential target for the development of therapeutic and preventive strategies for AD. In other words, maintenance of endogenous RSS homeostasis by daily intake of RSS-rich foods [19] or supplementation of exogenous RSS donors [25] could potentially be a target for AD prevention and therapy.

## Data Availability

Data are contained within the article and Supplementary Material.

## Supplementary Materials

The following supporting information can be downloaded at: https://www.mdpi.com/article/10.3390/antiox12051105/s1, Table S1: MRM parameters of NEM/TME-IAM adducts used for LC-ESI-MS/MS analyses; Figure S1. Determination of the cysteine content (A) and oxidized glutathione disulfide (GSSG): glutathione (GSH) ratio in the mouse cerebral cortex. Figure S2. Uncropped images of Western blotting for evaluation of protein polysulfides by a modified-switch assay using N-iodoacetyl L-tyrosine methyl ester (TME- IAM). Figure 5 shows the areas marked by red boxes. BM, biotin-maleimide.
