# Supplementary material for: Reactive Sulfur Species Omics Analysis in the Brain Tissue of the 5xFAD Mouse Model of Alzheimer’s Disease
Source: Antioxidants (Basel). 2023 May 16;12(5):1105. doi: 10.3390/antiox12051105 (PMC10215359; doi:10.3390/antiox12051105)
Supplement: Supplementary file 1 [file antioxidants-12-01105-s001.zip › antioxidants-2365750-supplementary.pdf]

Supplementary Materials

Reactive sulfur species omics analysis in the brain tissue of the 5xFAD mouse model of Alzheimer's disease

Ayaka Kinno[1], Shingo Kasamatsu[1], Takaaki Akaike[2], Hideshi Ihara[1, *]

[1]Department of Biological Chemistry Graduate School of Science, Osaka Metropolitan University, Sakai 599-8531, Osaka, Japan.

[2]Department of Environmental Medicine and Molecular Toxicology, Tohoku University Graduate School of Medicine, Sendai 980-8575, Miyagi, Japan.

*Corresponding author: Hideshi Ihara, Department of Biological Chemistry, Graduate School of Science, Osaka Metropolitan University, 1-1 Gakuen-cho, Sakai 599-8531, Osaka, Japan. Tel.: +81-72-254-9753; E-mail: iharah@omu.ac.jp

Supplementary Table S1. Multiple reaction monitoring (MRM) parameters of *N*-ethylmaleimide (NEM)/*N*-iodoacetyl L-tyrosine methyl ester (TME-IAM)-adducts used for liquid chromatography-electrospray ionization-tandem mass spectrometry (LC-ESI-MS/MS) analyses.

Analyte	Precursor ion (*m/z*)	Product ion (*m/z*)	Polarity	Cone voltage (V)	Collision energy (V)
NEM-S-NEM	285.1	126.1	+	35	20
NEM-^{34}S-NEM-d_{10}	297.1	131.1	+	35	20
BisS-AM-TME	505.5	136	+	35	40
BisS-AM-TME*	509.5	137	+	35	40
GS-AM-TME	543.2	414	+	35	20
GS-AM-TME*	545.2	416	+	35	20
GSS-AM-TME	575.5	446	+	40	20
GSS-AM-TME*	577.5	448	+	40	20
GSSG	613.1	355.1	+	30	20

NEM-S-NEM, bis-sulfur-NEM-adduct; BisS-AM-TME, TME-IAM-adduct of H_2S; GS-AM-TME, TME-IAM-adduct of glutathione; GSS-AM-TME, TME-IAM-adduct of glutathione hydropersulfide. GSSG, oxidized glutathione disulfide. *Stable isotope-labeled positions.

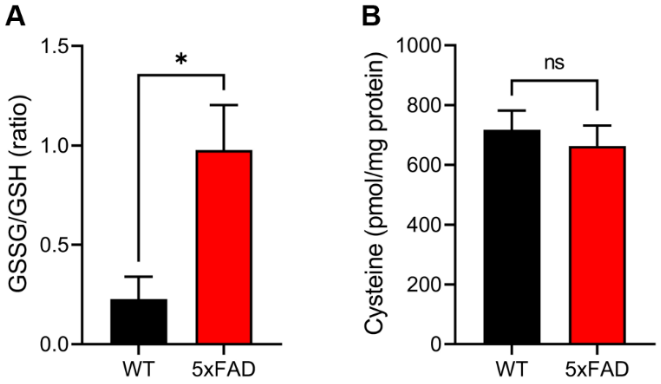

Supplementary Figure S1. Determination of the cysteine content (A) and oxidized glutathione disulfide (GSSG):glutathione (GSH) ratio in the mouse cerebral cortex. Data are presented as mean ± standard error (n = 3). *$p < 0.05$ versus the wild type (WT) group, compared by Student's unpaired t test. ns, not significant.
